# Genetic variation and recurrent parasitaemia in Peruvian *Plasmodium vivax* populations

**DOI:** 10.1186/1475-2875-13-67

**Published:** 2014-02-24

**Authors:** Andrea M McCollum, Valeria Soberon, Carola J Salas, Meddly L Santolalla, Venkatachalam Udhayakumar, Ananias A Escalante, Paul CF Graf, Salomon Durand, Cesar Cabezas, David J Bacon

**Affiliations:** 1 Malaria Branch, Division of Parasitic Diseases and Malaria, Center for Global Health, Centers for Disease Control and Prevention, Atlanta, GA, USA; 2 Atlanta Research and Education Foundation, Decatur, GA, USA; 3 Parasitology Programme, Naval Medical Research Unit No. 6, Lima, Peru; 4 Arizona State University, School of Life Sciences, Tempe, AZ, USA; 5 Instituto Nacional de Salud del Peru, Lima, Peru; 6 Current address: University of Rochester Medical Center, 601 Elmwood Ave, Rochester, NY 14642, USA; 7 Current address: Naval Research Laboratory, 4555 Overlook Drive, SW, Washington DC 20375, USA

**Keywords:** *Plasmodium vivax*, Malaria, Recrudescence, Relapse, Antigen locus, Microsatellite markers

## Abstract

**Background:**

*Plasmodium vivax* is a predominant species of malaria in parts of South America and there is increasing resistance to drugs to treat infections by *P. vivax*. The existence of latent hypnozoites further complicates the ability to classify recurrent infections as treatment failures due to relapse, recrudescence of hyponozoites or re-infections. Antigen loci are putatively under natural selection and may not be an optimal molecular marker to define parasite haplotypes in paired samples. Putatively neutral microsatellite loci, however, offer an assessment of neutral haplotypes. The objective here was to assess the utility of neutral microsatellite loci to reconcile cases of recurrent parasitaemia in Amazonian *P. vivax* populations in Peru.

**Methods:**

Patient blood samples were collected from three locations in or around Iquitos in the Peruvian Amazon. Five putatively neutral microsatellite loci were characterized from 445 samples to ascertain the within and amongst population variation. A total of 30 day 0 and day of recurrent parasitaemia samples were characterized at microsatellite loci and five polymorphic antigen loci for haplotype classification.

**Results:**

The genetic diversity at microsatellite loci was consistent with neutral levels of variation measured in other South American *P. vivax* populations. Results between antigen and microsatellite loci for the 30 day 0 and day of recurrent parasitaemia samples were the same for 80% of the pairs. The majority of non-concordant results were the result of differing alleles at microsatellite loci. This analysis estimates that 90% of the paired samples with the same microsatellite haplotype are unlikely to be due to a new infection.

**Conclusions:**

A population-level approach was used to yield a better estimate of the probability of a new infection versus relapse or recrudescence of homologous hypnozoites; hypnozoite activation was common for this cohort. Population studies are critical with the evaluation of genetic markers to assess *P. vivax* biology and epidemiology. The additional demonstration of microsatellite loci as neutral markers capable of distinguishing the origin of the recurrent parasites (new infection or originating from the patient) lends support to their use in assessment of treatment outcomes.

## Background

*Plasmodium vivax* contributes to a significant amount of morbidity in the Peruvian Amazon, accounting for 90% of all reported malaria cases in 2011 [1]. *P. vivax* is a predominant *Plasmodium* species in most areas of South America. The presence of the dormant hypnozoite stage along with reports of resistance to the most commonly used drugs to treat the blood stage, complicating treatment options for *P. vivax* complicate treatment options. Accordingly, the treatment and control of *P. vivax* remains an important issue to public health programmes.

*P. vivax* is characterized by the presence of a hypnozoite stage, which can persist in the liver for months to years, causing recurrent disease even though treatment and clearance of the initial blood stage infection were achieved. When a patient presents with recurrent *P. vivax* parasitaemia following treatment, there are several possible causes: 1) recrudescence of blood stage parasites; 2) relapse from latent hypnozoites; or, 3) a new infection (if the patient resides in an endemic area). Previous studies have shown that the risk and timing of relapse depends on the geographical origin of the initial infection. It is known that isolates of *P. vivax* from the tropics relapse sooner than those from more temperate areas, with 70% of relapses occurring within five months of initial infection [2]. The probability of relapse is higher than 20% for *P. vivax* patients when anti-hypnozoite therapy is not prescribed, and, thus, the ability to distinguish between relapse, recrudescence and re-infection is important in clinical efficacy studies [3].

Molecular genotype profiles of the parasites in an infection are critical to the assessment of clinical efficacy studies. These profiles may also be used to estimate the rate of new infections in populations that are continuously exposed. Increasingly, molecular genotyping has been used to differentiate treatment recrudescence from re-infection by comparing the profiles or haplotypes in a paired patient samples [4,5]. The probability that a given haplotype is identical in paired samples taken from two different time points greatly decreases with the use of a greater number of genetic loci and, also, with loci that have a high amount of polymorphism within a parasite population [6]. Using antigen-encoding genes is problematic as they are affected by immune selection. These genes alone or in concert are not able to distinguish recrudescence from re-infection with high levels of confidence [2].

Highly variable neutral microsatellite loci are ideal tools to assess population-level studies of *Plasmodium falciparum* and, more recently, *P. vivax*[7-16]. Complicating factors include the existence of multiple infections or clones within a single host and, in the case of *P. vivax*, the presence of dormant hypnozoites that persist for some time after the initial infection. However, studies that employ population level sampling and estimates of variation at each of the loci, in addition to paired patient samples taken at different points in time during an infection, can yield estimates of recurrence and drug failures [9,15]. An analysis of *P. vivax* populations and recurrent parasitemia was conducted. The haplotypes and the genetic variation allow for further assessment of paired patient samples as recrudescence/relapse or new infection.

## Methods

### Study sites and collection of samples

**Figure 1:**
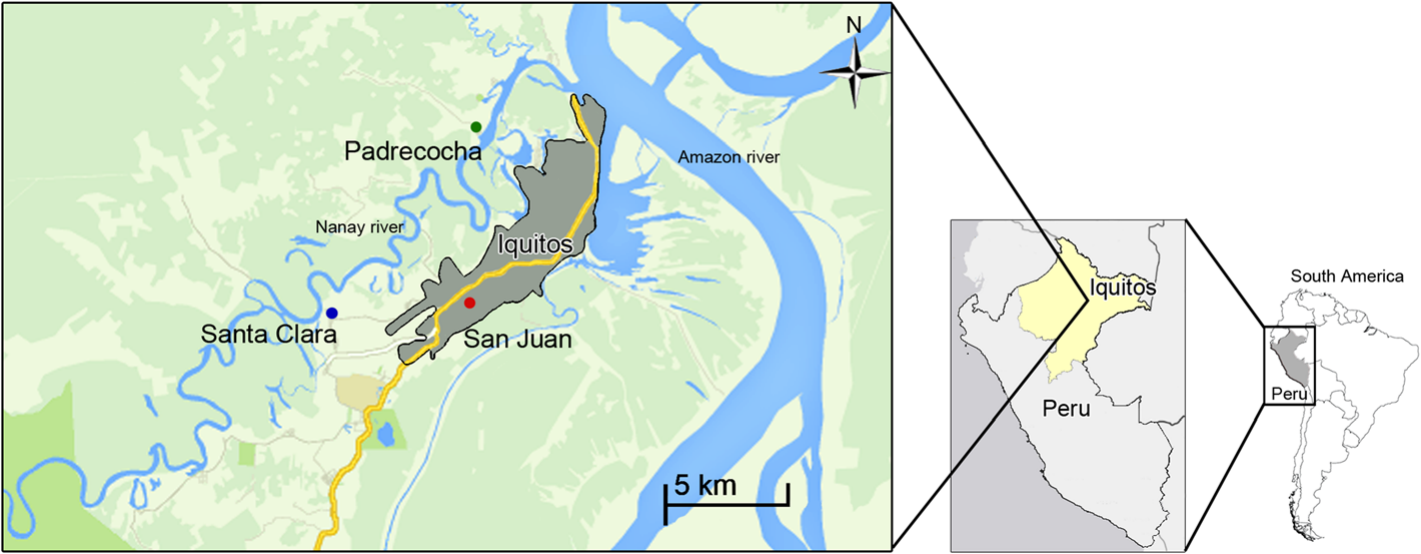
A map of the three populations used in this study, Padrecocha, Santa Clara, and San Juan.

Samples were collected under a study aimed at assessing the efficacy of three doses of primaquine for the prevention of *P. vivax* relapses. The study was conducted from March 2006 to November 2007 in three sites, Padrecocha, Santa Clara and San Juan, located in or near the city of Iquitos, the largest city in the Peruvian Amazon (Figure 1). Patients included in the study were at least one year of age, had a fever or history of fever within 72 hours, had a mono-infection with *P. vivax* as assessed by microscopy and PCR [17], had parasitaemia levels ranging between 250 and 100,000 asexual parasites/ul, were not pregnant, did not present with symptoms of severe malaria, and had normal activities of glucose-6-phosphate dehydrogenase (G6PD). All patients were treated under direct observation with chloroquine (25 mg/kg) over three days and three regimens of primaquine, depending on the study arm. Prior to receiving treatment, a pre-treatment whole blood sample was obtained on day 0 (D-0). Patients who presented a second time in the subsequent six months with recurrent *P. vivax* parasitaemia were treated with a second round of primaquine-chloroquine according to the protocol of the Peruvian Ministry of Health, and a second sample of whole blood was collected prior to re-treatment on the day of recurrence (D-R). All samples were stored at -80°C until they were used. Informed consent was granted by the subject or guardian in the case of minors and assent was obtained from subjects between eight and 17 years of age. The study protocol was approved by the Ethical Review Committees of the Peruvian National Institute of Health and the US Naval Medical Research Center in compliance with all applicable Federal regulations governing the protection of human subjects.

### DNA extraction

Parasitic DNA was extracted from 200 μl of whole blood following instructions from QIAamp DNA Blood Mini Kit (Qiagen). Genomic *P. vivax* DNA was used in order to amplify the polymorphic antigenic loci and also for the neutral microsatellite analysis.

### Antigen genotyping of *Plasmodium vivax*

Thirty paired samples (D-0 and D-R from the same subject) were genotyped for five polymorphic antigenic loci of *P. vivax*: apical membrane antigen-1 (*Pvama1*) [18], circumsporozoite protein (*Pvcsp*) [19], merozoite surface protein 1 (*Pvmsp-1*) [20], merozoite surface protein 3 (*Pvmsp3*) [21], and duffy receptor binding protein (*Pvdbp*) [22]. Products of single PCR reactions were sequenced using an Applied Biosystems Prism 3130xl Avant Genetic Analyzer, and data analysis was performed using Sequencer software (Gene Codes Corporation).

### Microsatellite characterization

Microsatellite characterization was conducted on 445 samples from 355 subjects (355 D-0 samples and 90 D-R samples) using five microsatellite loci in the *P. vivax* genome published previously by Imwong *et al.*: 14.185, 12.335, 7.67, 6.34, and 3.35 [10]. The full panel of eleven loci in the original study by Imwong *et al.*, were tested, but these five loci were chosen because they reliably produced a product on a multitude of specimens and spurious peaks were rarely detected. Further, these loci amplified a polymorphic loci in the Imwong *et al.*, study and also on a panel of test samples in the laboratory. The published PCR protocol was modified by using the PCR MasterMix (Promega) [10]. Fluorescently labelled PCR products were analysed on an Applied Biosystems Prism 3130xl Avant Genetic Analyzer and analysed using GeneMapper v4.0 (Applied Biosystems). A minimum peak height of 200 fluorescent units was used to define amplification products. For all analyses that use the microsatellite data, if multiple peaks were detected for a single sample, the value of the highest peak was used for the analysis.

### Statistical analysis

The genetic variation for each microsatellite locus was measured by calculating the expected heterozygosity (*H*_*e*_) and number of alleles per microsatellite locus (*A*). *H*_*e*_ was calculated for each locus as *H*_*e*_  =  [*n*/(*n* - 1)][1 - ∑ *p*_*i*_^2^], where *n* is the number of isolates sampled and *p*_*i*_ is the frequency of the *i*th allele. The sampling variance for *H*_*e*_ was calculated as 2(*n* - 1)/*n*^3^[2(*n* - 2)[∑ *p*_*i*_^3^  - (∑ *p*_*i*_^2^)^2^]] [23]. *H*_*e*_ and the sampling variance were calculated using D-0 samples only. Arlequin ver 3.01 was used to compute *H*_*e*_[24], and the Excel Microsatellite Toolkit was used to format data [25].

Wright’s fixation index, F_ST_, was used to test for genetic differentiation between two populations [26]. F_ST_ was calculated using Arlequin version 3.01 [24]. Linkage disequilibrium (LD) between microsatellite loci was assessed by using an exact test of LD [27]. Associations were tested between pairs of loci or sites by using 10,000 Monte Carlo steps in Arlequin; a Bonferroni-Holm correction was used to correct for multiple testing. For both F_ST_ and LD calculations, only microsatellite haplotypes from D-0 samples were used for the analyses. If a locus had multiple peaks, the allele represented by the highest peak was used for the analyses.

For paired D-0 and D-R samples, P(match), the probability of a second infection with a particular haplotype occurring purely by chance, was calculated using the population haplotype frequency. This calculation follows Brockman *et al.* and was conducted for the post-treatment sample pairs that had the same haplotype at each time point [28]. P(match) was calculated only for those sample pairs where the five-locus data was available; if data were missing from any of the loci then the paired samples were not considered for the P(match) analysis. Considering each geographic population separately, haplotype frequencies were determined using the five microsatellite loci and D-0 samples only. These frequencies were used as the ‘P(match)’ value for each of the haplotypes in question, and the P(match) value was considered significant if <0.05.

## Results

### Genetic relationships and differentiation among populations

In order to characterize the genetic variation overall and for each individual population, 355 D-0 samples were characterized at five neutral microsatellite loci. A total of 117, 54 and 184 samples were characterized for microsatellite loci from Padrecocha, Santa Clara, and San Juan, respectively. Approximately 2% of the samples in Padrecocha and San Juan and 3% in Santa Clara represented mixed infections of multiple *P. vivax* strains or haplotypes. *H*_*e*_, a measure of genetic variation per locus, was between 0.68 and 0.87 for each of the three populations individually and as a whole (Table 1). The loci 12.335, 7.67 and 3.35 had a higher number of alleles in each population and 14.185 and 6.34 had lower numbers of alleles.

**Table 1:** **Heterozygosity (*H***_***e***_**) and number of alleles (A) per microsatellite locus**

**Locus**	**14.185**		**12.335**		**7.67**		**6.34**		**3.35**	
	***H***_***e***_	**A**	***H***_***e***_	**A**	***H***_***e***_	**A**	***H***_***e***_	**A**	***H***_***e***_	**A**
Padrecocha	0.6833 ± 0.000	4	0.8430 ± 0.000	12	0.7149 ± 0.001	11	0.7711 ± 0.000	7	0.7998 ± 0.000	12
Santa Clara	0.7279 ± 0.001	6	0.8714 ± 0.000	11	0.7072 ± 0.002	9	0.7973 ± 0.000	7	0.8197 ± 0.001	10
San Juan	0.7282 ± 0.000	7	0.8665 ± 0.000	14	0.7227 ± 0.001	15	0.7775 ± 0.001	9	0.8071 ± 0.000	14
All	0.7173 ± 0.000	7	0.8665 ± 0.000	17	0.7168 ± 0.000	18	0.7812 ± 0.000	11	0.8149 ± 0.000	15

**Figure 2:**
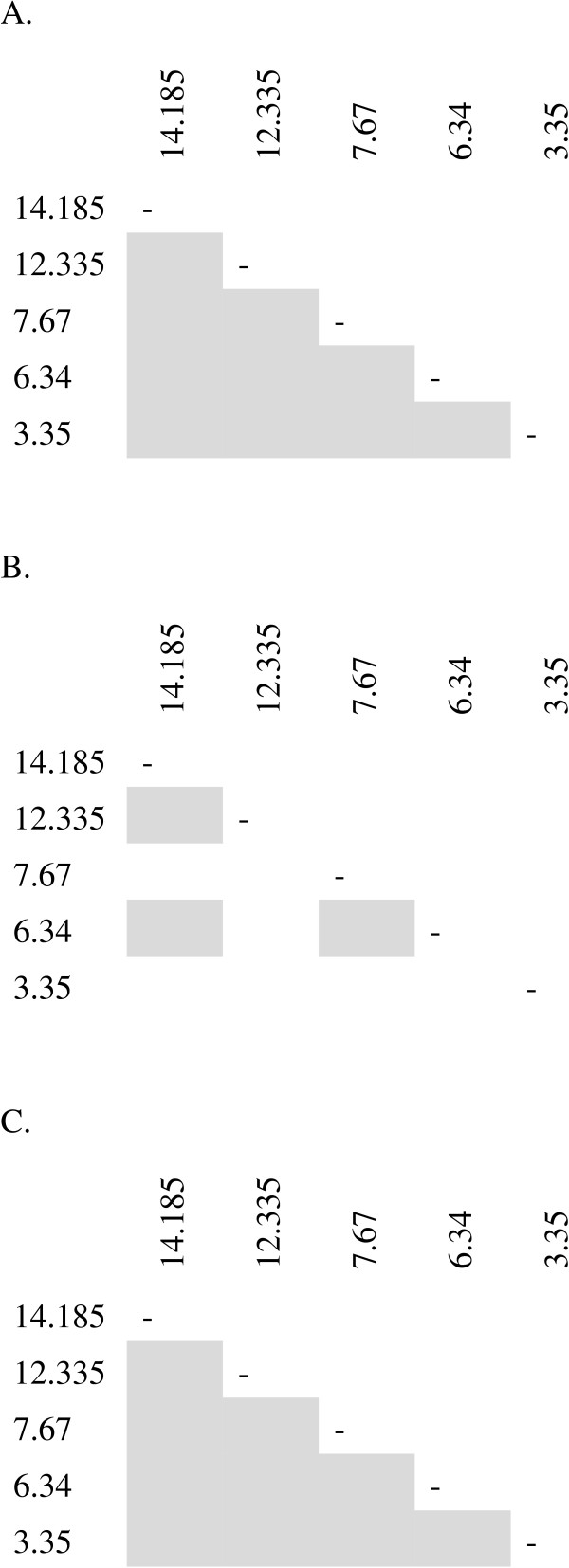
**Linkage disequilibrium between microsatellite loci at the three sites, Padrecocha (A), Santa Clara (B), and San Juan (C).** Each box represents one comparison between polymorphic pairs of loci. Shaded cells represent significance at 0.01.

There was a significant amount of genetic differentiation (measured by F_ST_) between Padrecocha and San Juan *P. vivax* populations (0.0118, p < 0.01). Additionally, the Padrecocha and San Juan *P. vivax* populations showed pairwise LD at all five loci. The Santa Clara population exhibited less LD (Figure 2).

### Analysis of paired samples

A total of 30 paired samples collected at the D-0 and D-R visits were analysed for *P. vivax* strain identity using five polymorphic antigenic loci (PAL): *Pvama*, *Pvcsp*, *Pvmsp1*, *Pvmsp3*, and *Pvdbp*. Twelve (40%) paired samples had identical antigenic haplotypes and 18 (60%) had at least one locus that differed between the paired samples (Table 2).

**Table 2:** Summary of polymorphic antigen loci (PAL) and microsatellite haplotypes from paired D-0 and D-R samples

	**Padrecocha**	**Santa Clara**	**San Juan**	**Total**
Number pairs with different^a^ PAL haplotype (%)	13 (65)	2 (100)	3 (37.5)	18 (60)
Number pairs with same^b^ PAL haplotype (%)	7 (35)	0 (0)	5 (62.5)	12 (40)
Number pairs with different^a^ microsatellite haplotype (%)	19 (55.9)	5 (50)	21 (45.7)	45 (50)
Number pairs with same^b^ microsatellite haplotype (%)	15 (44.1)	5 (50)	25 (54.3)	45 (50)
Number pairs where p(match) <0.05 (%)	9 (69)^c^	5 (100)	23 (100)^c^	37 (90.2)

^a^Different is defined as having at least one out of five loci different between paired samples.

^b^Same is defined as having identical alleles at all loci from which a viable PCR product was obtained.

^c^p (match) was only calculated for samples where there was complete data; two paired samples in both Padrecocha and San Juan populations had incomplete data.

Ninety paired D-0 and D-R samples were analysed for *P. vivax* strain identity by using the five loci microsatellite haplotypes (Table 3). Fifty percent of all pairs had identical alleles at all loci and the same proportion had a different allele at a minimum of one locus (Table 2). These proportions were similar for each of the three populations individually. A total of 37/41 paired samples (90.2%) had a significant P(match) < 0.05. Thus, it was unlikely that 90.2% of the paired samples were the result of a new infection. It should be noted that P(match) was only calculated for 41 out of 45 total pairs where the haplotypes were the same. Four pairs had incomplete data, and thus were removed from P(match) calculations.

**Table 3:** Microsatellite analysis of paired D-0 and D-R samples

**Population**	**Case**	**Treatment**^**a**^	**Day**^**b**^	**14.185**^**c**^	**12.335**	**7.67**	**6.34**	**3.35**	**MS final**^**d**^	**p(match)**^**e**^
Padrecocha	1	5	0	269	162	102	150	125	S	0.01
			70	269	162	102	150	125		
	2	5	0	269	160	100	135	127	D	
			183	269	166	100	160	127		
	3	5	0				135		D	
			141				160			
	4	7	0	265	171	102	150		S	
			84	265	171	102	150			
	5	14	0	265	171		135		D	
			83	265	166		135			
	6	5	0	269	158	127	146	125	D	
			154	269	158	127	150	151		
	7	5	0	282	162	100	146		D	
			105	265	171	102	150			
	8	14	0	282	166	100	135	135	D	
			81	265	171	102	150	151		
	9	7	0	282	166		160		D	
			28	282	166		135			
	10	7	0	282	162	101	150	151	D	
			43	282	166	101	135	133		
	11	5	0	282	163	98	150	151	D	
			120	282	163	98	150	153		
	12	14	0	282	166		135	133	D	
			55	282	166		160	127		
	13	5	0	269	158	126	146	125	D	
			154	269	158	126	135	127		
	14	5	0	269	158	125	145	125	S	0.051
			210	269	158	125	145	125		
	15	14	0	282	166		160	125	D	
			43	282	166		135	133		
	16	7	0	282	166	102	135	133	D	
			105	269	162	102	160	125		
	17	5	0	282	166	122	150	117	D	
			53	282	166	101	135	125		
	18	5	0	269	166	101	160	127	D	
			63	282	162	101	146	125		
	19	14	0	265	171	102	150	153	S	0.02
			91	265	171	102	150	153		
	20	5	0	265	171	102	150	153	D	
			84	265	171	102	146	125		
	21	5	0	269	158	125	145	125	S	0.031
			111	269	158	125	145	125		
	22	5	0	269	158	125	145	125	S	0.031
			126	269	158	125	145	125		
	23	14	0	269	159	100	135	127	S	0.01
			73	269	159	100	135	127		
	24	5	0	265	170	102	150	153	D	
			70	265	170	102	150	125		
	25	7	0	265	170	101	150	153	S	0.01
			53	265	170	101	150	153		
	26	7	0	265	170	102	149	153	S	0.051
			76	265	170	102	149	153		
	27	14	0	265			149	153	D	
			52	265			134	131		
	28	5	0	265	170	102	149	153	S	0.051
			112	265	170	102	149	153		
	29	7	0	265	170	102	149	153	S	0.051
			80	265	170	102	149	153		
	30	14	0	265	170	102	149	153	S	0.051
			67	265	170	102	149	153		
	31	7	0	265	170	102	149	153	S	0.051
			120	265	170	102	149	153		
	32	5	0	265			136	125	D	
			63	265			134			
	33	5	0	265	170	102	150	153	S	0.01
			202	265	170	102	150	153		
	34	7	0	265			149		S	
			121	265			149	153		
Santa Clara	35	7	0	265	162	98	150	151	D	
			52	265	164	102	135	127		
	36	5	0	265	162	100	135	119	D	
			126	265	162	100	135	127		
	37	5	0	265	171	102	150	153	D	
			87	265	171	102	150	151		
	38	14	0	265	164	101	150	135	D	
			134	265	164	101	150	125		
	39	5	0	265	170	102	149	153	S	0.019
			30	265	170	102	149	153		
	40	5	0	265			160	125	D	
			38	265			160	153		
	41	14	0	265	158	125	145	125	S	0.019
			178	265	158	125	145	125		
	42	5	0	265	158	126	145	125	S	0.019
			117	265	158	126	145	125		
	43	5	0	265	170	102	149	127	S	0.019
			110	265	170	102	149	127		
	44	14	0	265	164	115	149	125	S	0.019
			32	265	164	115	149	125		
San Juan	45	7	0	265			150		D	
			28	265			135			
	46	5	0	265	162	102	150	127	D	
			43	265	171	102	150	151		
	47	5	0	265	166	122	150	127	S	0.006
			71	265	166	122	150	127		
	48	7	0	265	166	122	150	117	D	
			40	265	164	104	135	125		
	49	5	0	265	164	102	135		D	
			62	265	164	102	135			
	50	14	0	265	158	102	135	127	D	
			84	265	171	102	150	127		
	51	5	0	265	171	102	150	127	S	0.006
			70	265	171	102	150	127		
	52	5	0	265	171	102	150	151	S	0.006
			80	265	171	102	150	151		
	53	14	0	265	164	101	160	125	S	0.012
			99	265	164	101	160	125		
	54	14	0	265	158	125	149	125	D	
			144	265	158	125	145	125		
	55	5	0	265	160	102	145	151	D	
			86	265	160	102	145	151		
	56	5	0	265	162	102	135	151	S	0.006
			51	265	162	102	135	151		
	57	5	0	265	170	100	150	127	S	0.006
			53	265	170	100	150	127		
	58	5	0	265	174	100	134	119	S	0.006
			53	265	174	100	134	119		
	59	7	0	265	160		160	125	S	
			120	265	160		160	125		
	60	5	0	265	162	101	150	149	S	0.006
			72	265	162	101	150	149		
	61	7	0	265			134		D	
			99	265			134			
	62	7	0	265	170	100	159	153	S	0.006
			129	265	170	100	159	153		
	63	5	0	265	174	102	134	125	S	0.006
			42	265	174	102	134	125		
	64	5	0	265			149	121	D	
			122	265			159	121		
	65	14	0	265	166	101	134	121	S	0.006
			98	265	166	101	134	121		
	66	14	0	265	174	100	149	133	D	
			56	265	170	102	149	133		
	67	14	0	265	166	151	134	127	S	0.006
			100	265	166	151	134	127		
	68	5	0	265	158	122	149	127	D	
			113	265	158	122	149	127		
	69	5	0	265			159	121	D	
			85	265			149	121		
	70	14	0	265	166	121	134	121	S	0.006
			76	265	166	121	134	121		
	71	5	0	265		101	145		D	
			210	265	164	105	150			
	72	5	0	265	162	100	160	153	S	0.006
			162	265	162	100	160	153		
	73	7	0	265	166	99	135		D	
			71	265	162	121	149			
	74	5	0	265	164	101	160	125	S	0.012
			103	265	164	101	160	125		
	75	5	0	265	166	101	134		D	
			197	265	162	101	134			
	76	5	0	265	170	102	150	125	S	0.006
			72	265	170	102	150	125		
	77	5	0	265	166	100	160	125	S	0.006
			56	265	166	100	160	125		
	78	5	0	265			135	133	D	
			168	265			135	148		
	79	14	0	265	158	125	134	127	D	
			80	265	154	125	134	151		
	80	7	0	265	170	101	149	125	D	
			51	265	166	121	149	125		
	81	5	0	265			134		S	
			136	265			134	121		
	82	5	0	265			145		D	
			59	265			135	151		
	83	5	0	265	174	102	149	133	D	
			163	265	170	102	149	148		
	84	7	0	269	162	103	145	153	S	0.006
			168	269	162	103	145	153		
	85	14	0	269	162	100	149	148	S	0.006
			71	269	162	100	149	148		
	86	14	0	265	167	101	149	153	S	0.006
			98	265	167	101	149	153		
	87	5	0	269	162	121	149	127	S	0.006
			136	269	162	121	149	127		
	88	5	0	269	166	101	135	153	S	0.006
			79	269	166	101	135	153		
	89	14	0	271	166	101	134	121	D	
			183	276	162	100	140	148		
	90	14	0	282	162	100	160	153	S	0.006
			73	282	162	100	160	153		

^a^Treatment given to the patient on D-0. ‘5’ represents 30 mg of primaquine for 5 days; ‘7’ represents 30 mg of primaquine for 7 days.

‘14’ represents 15 mg of primaquine for 14 days.

^b^Day of sample collection, where ‘0’ is Day-0 (D-0) and the second day is the day of recurrence (D-R) respective to D-0.

^c^Allele for each microsatellite loci are reported as PCR product size.

^d^The final determination of concordance (S = same) or disconcordance (D = different) between the two microsatellite haplotypes (D-0 and D-R). Different alleles between a pair of samples are highlighted in red.

^e^P(match) values were calculated separately for each population and only for complete 5-locus haplotypes.

The microsatellite data were compared to the PAL data for 30 paired samples. Concordance between the paired samples was examined for both PAL and microsatellite data (both having the criteria of no differing loci for a D-0 and D-R pair). Twenty-four pairs (24/30, 80%) had concordant results between the two methods and six pairs (six/30, 20%) were non-concordant. Of the six that were non-concordant, one pair was determined as the same haplotype by microsatellite analysis at both time points and a different haplotype by antigen loci (the difference was at one of five loci, *Pvcsp*). Five pairs were found to have the same haplotype by antigen loci but a different haplotype by using microsatellite loci.

## Discussion

The high levels of genetic diversity seen in these samples from the Peruvian Amazon are consistent with neutral levels of microsatellite and tandem repeat variation measured in *P. vivax* from multiple sites in Brazil, Colombia, and Peru [7,13,15,29-32]. This pattern is curious because it is entirely different from South American *P. falciparum* populations, as they show much lower levels of variation [33]. These differences could be explained by a number of factors, including differences in demography, natural selection due to drug pressure or differences in microsatellite dynamics between the two *Plasmodium* species. A low number of multiple clone infections in individual patients were observed in this study. This is an area where there is relatively lower transmission of parasites, and it is expected that these patients would experience less inoculations than patients in a hyperemic area of disease, and, thus a reduced chance for multiple clones existing within a single patient. Further, the specific composition of loci examined (i.e. the microsatellite motifs) and the effects of natural selection (i.e. strong immune selection on PALs) on these loci, may affect the detection of multiple infections [34].

There was evidence of population differentiation between Padrecocha and San Juan; this differentiation was not observed between Santa Clara and either of the two sites. This result could be due to a sample size bias, as only 54 samples were available from Santa Clara. Extensive LD among microsatellite markers has been noted in Colombian and some (but not all) Brazilian *P. vivax* populations. The existence of LD in South American populations is not surprising, given the low rate of parasite transmission and high levels of genetic inbreeding documented in many areas of the continent [13,29,35]. The Santa Clara population had less observed LD, but this population was represented by a smaller number of samples, which could affect the calculation of LD as it is dependent on the frequency of allele or loci pairs in a population. An alternative explanation could be that the Santa Clara population has experienced a larger amount of genetic recombination or mutation. Additional studies would be needed to better understand this observation.

There have been several studies that have utilized population-based estimates of genetic variation to calculate the probability that a pair of samples from the same patient would have the same genetic profile or haplotype by chance. P (identical), the probability that a random pair of haplotypes is identical, is affected by low frequency haplotypes in a population [36]. There are many low frequency haplotypes in each of the three Peruvian populations evaluated here. Another estimate, p (maximum), the maximum probability of two clones carrying the same haplotype by chance, requires independence of loci [9]. There was extensive LD between loci in the populations from Padrecocha and San Juan; thus, non-independence of loci was found. The third method, P(match), utilizes the population haplotype frequencies to assess whether the paired haplotype frequency occurs above or below a defined frequency value (for example, 0.05) [28]. Given the before mentioned limitations of the populations, P(match) was chosen as a method to assess the maximum probability of two clones or parasites carrying the same haplotype by chance in a population. P(match) has the limitation of requiring a population-level assessment of parasite diversity prior to assigning probability values. In areas with low genetic diversity, as is observed in South America, sample sizes must be relatively large in order to appropriately define the extent of diversity in the parasite populations [28].

Eighty percent of the paired samples examined by both microsatellite and antigen loci methods obtained the same overall result. Five out of six non-concordant paired samples (83%) were found to have different haplotypes at the two sampling times by microsatellite loci, but not by using antigen loci. This result is not unexpected, as microsatellite loci are putatively neutral with respect to selection, and, thus, should exhibit a greater amount of variation. Loci under strong selection, such as antigen loci, exhibit lower levels of variation in the population. These results emphasize the utility of using neutral loci to determine the *P. vivax* strain characteristics, and this can be illustrated by considering the application of these tests to assess a treatment regimen. If, for example, this study assessed drug failures amongst these 30 paired samples, results from antigen-encoding loci would lead one to believe that 12 (40%) patients potentially had a relapse of liver-stage parasites, whereas microsatellite loci would assess this rate at eight (26.7%) patients. It should be emphasized, though, that this type of simple interpretation has many caveats. Of note, the microsatellite methods used here employed a population-based level of sampling and analysis - such an approach is critical when assessing and interpreting genetic variation. Although use of PAL and microsatellite loci can be used in conjunction, researchers should use caution in the interpretation of the data and consider the effect of selection or neutral mechanisms on each of type of loci.

Interpretation of recurrent parasitaemia and drug treatment regimens in *P. vivax* is complicated by the presence of hypnozoites. Although the transmission of *P. vivax* in the Iquitos area of Peru is relatively low, the possibility of re-infection for the subjects in this study cannot be eliminated. Paired samples with different initial and recurrent haplotypes are the result of relapse with a heterologous hypnozoite or a new infection; here, 50% of the paired samples had different parasite lineages. Paired samples with the same lineage (here, 50%) are the result of recrudescence of the blood stage parasite (potentially a chloroquine treatment failure), relapse with a homologous hypnozoite (potentially a primaquine treatment failure), or a new infection with the same parasite strain. Furthermore, the population-level analysis here indicates that 90.2% of the paired samples that had the same haplotype were unlikely due to a new infection. These results indicate a potential large frequency of recurrent infections resulting from hypnozoite activation, either heterologous or homologous. This could arise from either an initial infection or multiple infections over time with different *P. vivax* strains, all resulting in dormant hypnozoites. The activation of heterologous hypnozoites has been noted before in patients from Asia [9].

There are limitations to microsatellite, tandem repeats, and PCR-based techniques to discern treatment outcomes. PCR bias, potential artifacts, and the inability to discriminate multiple clones are significant hurdles with these methods [14]. Indeed, microsatellite diversity is associated with the underlying repeat motif structure of each loci, and, thus, there may be an inherent bias in the results based on the loci that are chosen [32,34,37]. Careful consideration of the underlying motif structure of these loci is an important factor for researchers to consider. Further, the ability to rapidly screen many infections and assess genetic variation of the larger parasite population is key to understanding and interpreting these assays, irrespective of the locus. Population-based approaches have an advantage over smaller sampling strategies in both analyses and assessments [15].

In conclusion, the *P. vivax* parasite population in the Peruvian Amazon has considerable diversity and population differentiation. Antigen and microsatellite-based *P. vivax* genotyping methods can be useful tools to evaluate drug treatment regimens and characterize genetic profiles of parasite populations; however, researchers should be careful when interpreting data. Antigen-encoding loci do incorporate a bias due to immune selection. The description of the extent of hypnozoite activation in this population emphasizes the importance of treatments that target this stage. The *P. vivax* dormant hypnozoite stage poses challenges in the assessment of paired patient samples; nevertheless, population-based studies with appropriate methods can provide insights into the biology and epidemiology of *P. vivax* populations. The further development and assessment of molecular tools that allow a broad analysis of an individual parasite’s genetic construction will be immensely useful to drug treatment and resistance studies.

## Competing interests

The authors declare that they have no competing interests.

## Authors’ contributions

AMM, VU, AAE, and DJB designed the study. AMM, VS, CS, and MS conducted the molecular genetics studies and data analysis. AMM, PCFG and DJB drafted the manuscript. SD, CC and DJB participated in the field study design and coordination of sample collection. All authors read and approved the final manuscript.

## Copyright statement

Some of the authors are a military service members or employees of the U.S. Government. This work was prepared as part of their official duties. Title 17 U.S.C. §105 provides that ‘Copyright protection under this title is not available for any work of the United States Government.’ Title 17 U.S.C. §101 defines a U.S. Government work as a work prepared by a military service member or employee of the U.S. Government as part of that person’s official duties.
